# The prospective study of cardiac MRI in the diagnosis and treatment of cardiac sarcoidosis associated with atrioventricular block

**DOI:** 10.1186/1532-429X-17-S1-P373

**Published:** 2015-02-03

**Authors:** Satoshi Kunimoto, Kensuke Yamamoto, Toshiko Nakai, Yasuo Okumura, Mahoto Kato, Tadateru Takayama, Takafumi Hiro, Ichiro Watanabe, Atsushi Hirayama

**Affiliations:** 1 Division of Cardiology, Nihon University School of Medicine, Tokyo, Japan

## Background

Sarcoidosis is systemic granulomatous disease of unknown etiology. Most patients present with pulmonary involvement, but association of cardiac sarcoidosis (CS) is critical factor determining prognosis. Recently, cardiac magnetic resonance imaging (CMR) and 18F-fluoro-2-deoxyglucose positron emission tomography (18F-FDG PET) can detect cardiac lesions in asymptomatic sarcoidosis patients. On the other hand, Conduction abnormalities and ventricular tachycardia are the most common arrhythmias with CS. With the experience of CS pointed out accidentally in PET medical examination at earlier stage got us to recognize the usefulness of CMR, we conducted a study to figure out real-world prevalence of CS in advanced atrioventricular block (AVB) cases and to assess the usability of "aggressive" diagnosis of CS by cardiac MRI.

**Figure 1:**
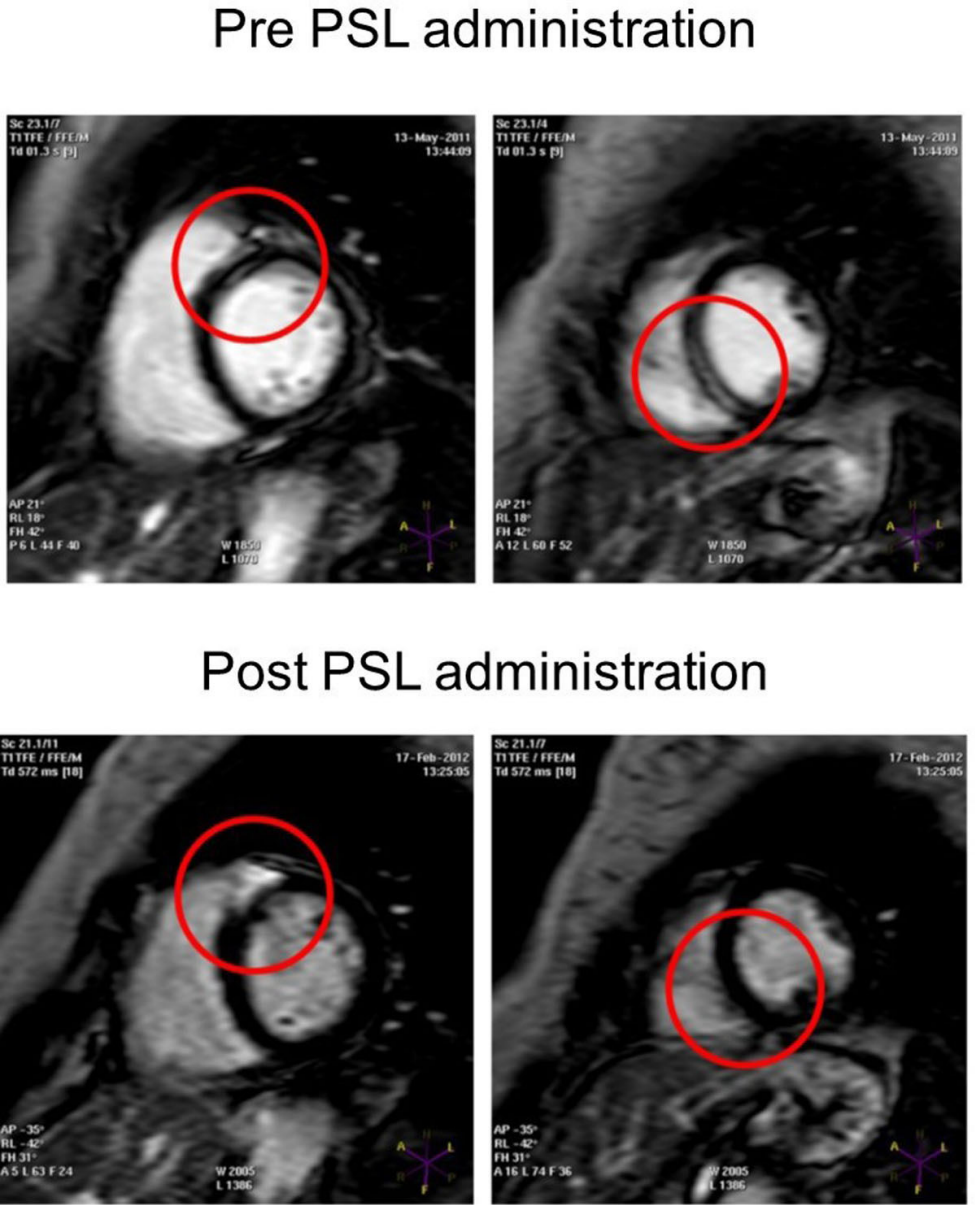
The effect of PSL administration to cardiac sarcoidosis, which prevent pacemaker implantation.

## Methods

Between June 2009 and May 2013, one thousand four hundred eighty-seven patients (nine hundred fifty-two males) underwent CMR. We underwent CMR to advanced AVB cases that have been the adaptation of pacemaker implantation of five years if at all possible. CMR was performed by using 1.5-T magnet MR systems (Intera Achieva, Philips Medical Systems) with 5-element cardiac coils. In cases with temporary pacing, CMR scan were performed just behind extraction temporary pacing lead with confirmation of spontaneous beat, which followed by pacemaker implantation. We excluded cases over 85 years old or in severe circulatory failure with AVB.

## Results

Sixty-six AVB cases of all fourteen hundred eighty-seven CMR cases underwent CMR for five years. On the other hand, in one hundred fifty-four cases, a pacemaker was newly implanted for AVB and one hundred twenty-five were under 85 years old. Among these cases, CMR scan were performed in fifty (40% of one hundred fifty-five) cases. Thirteen cases (20% of sixty-six) indicate the pattern of CS with late Gd enhancement (LGE). Nine of thirteen cases were diagnosed as CS with criteria and FDG-PET scan. Five cases received steroid therapy and three cases showed improvement of AVB. Especially, pacemaker implantation was able to avoid in one case. Unfortunately one case did not receive steroid therapy at early phase and developed congestive heart failure. In addition, FDG-PET is more useful than Ga scintigram for assessment activity of CS.

## Conclusions

CMR in advanced AVB has been accompanied with difficulty because of the bradycardia. Even if only few cases were found as CS in all cases newly pacemaker implanted, its impact on the therapeutic strategy is enormous. In diagnosis of CS, sign of positive LGE and positive PET have important implications. CMR is useful as screening tool for CS and should be performed as far as possible in advanced AVB cases.

## Funding

N/A.

